# Simultaneous induction of Graves’ hyperthyroidism and Graves’ ophthalmopathy by TSHR genetic immunization in BALB/c mice

**DOI:** 10.1371/journal.pone.0174260

**Published:** 2017-03-20

**Authors:** Nan Xia, Xiaozhen Ye, Xiaohao Hu, Shiyu Song, Hui Xu, Mengyuan Niu, Hongwei Wang, Jian Wang

**Affiliations:** 1 Department of Endocrinology, Jingling Hospital, Medical School of Nanjing University, Nanjing, P.R. China; 2 Center for Translational Medicine and Jiangsu Key Laboratory of Molecular Medicine, Medical School of Nanjing University, Nanjing, P.R. China; 3 Department of Endocrinology, Mingci Cardiovascular Hospital, Wuxi, P.R. China; University of Leicester, UNITED KINGDOM

## Abstract

**Background:**

Graves’ disease is the most common form of autoimmune thyroid disorder, characterized by hyperthyroidism due to circulating autoantibodies. To address the pathological features and establish a therapeutic approach of this disease, an animal model carrying the phenotype of Graves’ disease (GD) in concert with Graves’ Ophthalmopathy (GO) will be very important. However, there are no ideal animal models that are currently available. The aim of the present study is to establish an animal model of GD and GO disease, and its pathological features were further characterized.

**Methods:**

A recombinant plasmid pcDNA3.1- T289 was constructed by inserting the TSHR A-subunit gene into the expression vector pcDNA3.1, and genetic immunization was successfully performed by intramuscular injection of the plasmid pcDNA3.1-T289 on female 8-week-old BALB/c mice. Each injection was immediately followed by *in vivo* electroporation using ECM830 square wave electroporator. Morphological changes of the eyes were examined using 7.0T MRI scanner. Levels of serum T4 and TSHR antibodies (TRAb) were assessed by ELISA. The pathological changes of the thyroid and orbital tissues were examined by histological staining such as H&E staining and Alcian blue staining.

**Results:**

More than 90% of the immunized mice spontaneously developed goiter, and about 80% of the immunized mice manifested increased serum T4 and TRAb levels, combined with hypertrophy and hyperplasia of thyroid follicles. A significantly increased synthesis of hyaluronic acid was detected in in the immunized mice compared with the control groups.

**Conclusion:**

We have successfully established an animal model manifesting Graves’ hyperthyroidism and ophthalmopathy, which provides a useful tool for future study of the pathological features and the development of novel therapies of the diseases.

## Introduction

Graves’ disease (GD) is an organ specific autoimmune disease, characterized by the presence of autoantibodies directed against the thyrotropin receptor (TSHR). The pathological features can be manifestd as hyperthyroidism, diffuse goiter, Graves’ ophthalmopathy (GO) and pretibial myxedema. It is generally believed that TSHR stimulating antibodies (TSAb) are the major cause for the induction of a large amount of thyroid hormones through activation of cAMP signaling pathway. Excessive release of thyroid hormone results in the clinical manifestations of hyperthyroidism including goiter, weight loss, hyperactivity, nervousness, irritability and a sense of easy fatigability etc. [1]. About 30%-60% GD patients present with symptoms of GO such as a dry and gritty ocular sensation, photophobia, excessive tearing, and double vision. There are 3%-5% GO patients who suffer from severe outcomes such as corneal ulceration, compressive optic neuropathy or even loss of sight. GO is also characterized by soft tissue swelling and apoptosis, a result of increased adipose tissue and excessive production of glycosaminoglycans (GAGs, mainly hyaluronan) in retrobulbar tissue. Histopathological examination also show infiltration of immune cells in retrobulbar tissue, including T cells, B cells, mast cells and macrophages[2].

However, the study of the pathological mechanism of GD and GO are hampered by the lack of a universal animal model. Since there are no spontaneous mouse model of GD and GO available, attempts to increase the expression of TSHR in vivo for the establishment of GD and GO has attracted a lot of attention, but at the same time exhibited various different results [3]. Among these attempts, genetic immunization using adenovirus vectors in female BALB/c mice has been reported to be able to induce the phenotype of GD [4, 5]. In one recent study, immunization with adeno-TSHR289 has induced a long-term and steady murine model of Graves’ like disease and also the orbital manifestations[6]. However, genetic immunization via electroporation has the merit of not involving unnecessary antigens derived from the cell lines or virus and it does not require the time for establishing a cell line expressing human TSHR(hTSHR). Moreover, in recent years, a study from Moshkelgosha and colleagues has reported the establishment of GO model by immunizing hTSHR A subunit expressing recombinant plasmid [7, 8]. This method was proved to be very effective by inducing the GO murine model, however, this model is not completely ideal since the immunized animals exhibit hypothyroidism rather than hyperthyroidism, which is not the most common thyroid manifestations in GO patients. In the present study, by modifying the experimental protocol, we have developed a new approach of genetic immunization by intramuscular (i.m) injection of hTSHR A subunit expressing recombinant plasmid, which was found to be able to induce a steady and repeatable murine model of GD in concert with GO. Therefore, our study provided a useful approach for investigation of the pathological features and development of the therapeutic methods of the diseases in the future.

## Materials and methods

The experimental procedures performed on mice were conducted in accordance with the approved guidelines in the ethical permit approved by the Nanjing University Animal Welfare and Ethics committee.

### Construction of human TSHR A subunit expressing recombinant plasmids

*Human TSHR A subunit* cDNA (amino acid residues 1–289) was obtained directly by artificial gene synthesis (*NM_000369*.*2*). Then, the DNA fragment was digested with the BamHI and XhoI enzyme before subcloned into pcDNA3.1 (+) vector to generate the recombinant plasmid, which was designated as pcDNA3.1- T289. The constructs were then verified by restriction enzyme digestion and confirmed by DNA sequencing.

### Mice and immunization

Female BALB/c mice aged 6–8 weeks were purchased from Model Animal Research Center of Nanjing University (Nanjing, China) and were housed under specific pathogen-free conditions. Mice were allowed free access to food and water and were used in accordance with the standard of Animal Research Ethics Committee of Medical School of Nanjing University. We have performed the experiment twice and each time the mice were randomly divided into 3 groups: n (T289) = 8, n (Control) = 4, and n (pcDNA 3.1) = 4. All mice were anesthetized by injecting intraperitoneally sodium pentobarbital (40mg/kg), after which they received intramuscular (i.m) immunization of plasmid DNA (100 mg/mouse) of pcDNA3.1-T289, pcDNA3.1(+), or saline as the mock control. The intramuscular (i.m) immunization was peformed in the biceps femoris muscle. The depth of needles was around 4-5mm inside the muscle. Injections were immediately followed by electroporation with an ECM 830 system (BTX Harvard Apparatus, USA) with 10mm electrode needles at 200 V/cm. The parameters for electroporation were as previously described [8]. The current was applied in ten 20 ms square wave pulses at 1 Hz and caused marked muscle twitching. The injections were performed four times in total at 3-week intervals [4]. Animals were monitored for signs of distress afterwards, including weight loss and behavior changes such as fur-ruffling and lethargy. No animal was ill or died during the experiment. 2 weeks after the last immunization, all mice were humanly sacrificed and blood, thyroid and spleen were collected. Serum samples were stored at -80°C until assayed for total serum thyroxine (T4) and TSHR antibodies (TRAb).

### MRI scanning

One week after the last immunization, mice were subjected to 7.0T MRI scanning for the examination of orbit and extraocular muscle (the eyes and the frontal region of the brain). All parameters were set after mice were anesthetized [8]. Respiration and heart rate were monitored throughout the entire scanning process. The images were analyzed using Image J software and segmentation analysis was used to calculate the volume of extraocular muscles.

### Measurement of serum antibodies and thyroid function

Serum T4 and TRAb levels were both determined by enzyme-linked immunosorbent assay (ELISA). Serum T4 was tested using mouse T4 ELISA kit (ExCell, Shanghai, China) according to the instructions. TRAb levels in sera were measured using ELISA following the methods as previously described [9]. Briefly, 96 well plates were coated overnight with 100 μl of purified TSHR (NT) protein (Yubo biotech. Shanghai, China) which was diluted to 100 μg/ml in PBS at 4°C. Free protein binding sites were blocked by adding 1% BSA for 2 hours at 37°C. Duplicate aliquots of sera diluted 1:1000 were analyzed and Ab binding was detected with horseradish peroxidase—conjugated mouse anti-IgG (Sigma-Aldrich, USA). The OD value was read at 450 nm. The TRAb levels were expressed as the fold change of OD450 value of sample OD compared to control sera.

### Histological examination of thyroid and orbit tissues

Thyroid—trachea preparations and the entire orbital bony tissues (including the orbital bones with the eyeball, extraocular muscles, and the optic nerve) were collected and fixed in 4% paraformaldehyde[10]. The orbital bony tissue was placed in 10% EDTA decalcification solution for at least 15 days with two changes of the solution. Thyroids and orbital tissues were all embedded in paraffin, and sectioned for hematoxylin and eosin (H&E) staining. In addition, Alcian blue staining was performed on orbital sections for detection of hyaluronic acid (HA) following the protocols as described previously[11, 12].

### Statistical analysis

Statistical analysis was performed by GraphPad Prism 6.0 (GraphPad Software Inc.). Data was analyzed using the t test and one-way ANOVA. MR images were analyzed using Image J software (NIH) and the extraocular muscle volumes are measured by segmentation analysis, P-values < 0.05 represent significant differences.

## Results

### Construction of the recombinant plasmid and expression of TSHR A subunit gene in eukaryotic cells

By blasting the consensus sequence in GenBank, the constructed DNA sequence consensus contained 100% matching residues compared with published *human TSHR A subunit* gene sequence (*NM_000369*.*2*).

**Fig 1:**
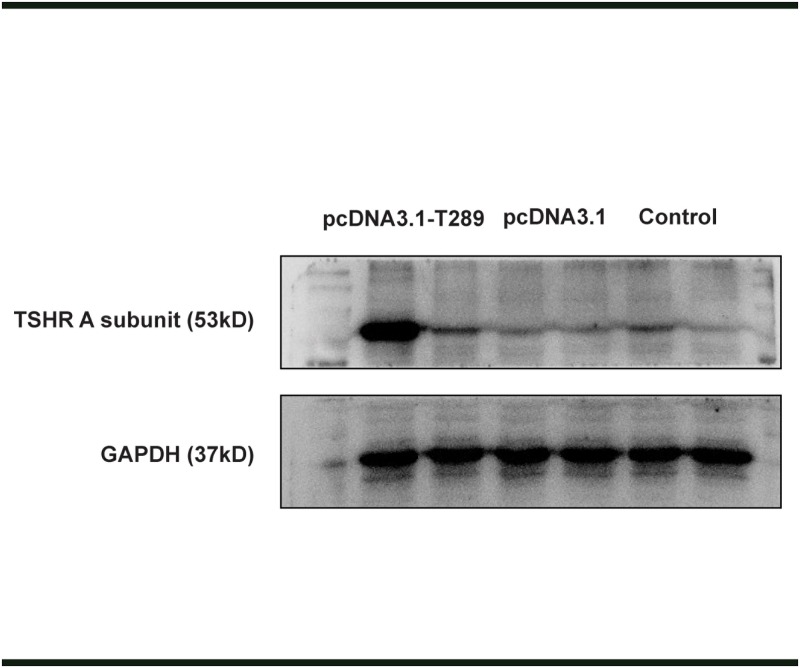
Construction of recombinant plasmid and the expression of human TSHR A subunit via transfected NIH 3T3L1 cells. Identification of TSHR gene expression by western blot. Cultured 3T3L1 cells were transfected with the recombinant plasmids pcDNA3.1- T289 or the control vector pcDNA3.1 using Lipofectamine 3000 (Invitrogen). 24h after the transfection, western blot assay was performed to detect the expression of TSHR A subunit protein. The first lane of pcDNA3.1-T289 showing more enhanced expression than the second one was due to the different transfection efficiency in different cell plates.

To determine whether the recombinant plasmid could express TSHR A subunit protein, we transfected pcDNA3.1- T289 plasmid DNA into 3T3L1 cells, as compared, pcDNA3.1 plasmids DNA were transfected as a mock control. As shown in Fig 1, increased expression of TSHR A subunit was detected in pcDNA3.1- T289 transfected cells, which is visible with a molecular weight of approximately 62kDa. In contrast, there was no specific band in the negative control group or the pcDNA3.1-transfected cells. These results indicated that we have successfully constructed a recombinant plasmid and the plasmid could successfully express as the corresponding protein in eukaryotic cells after *in vitro* transfection.

### TSHR genetically immunized mice developed hyperthyroid phenotype

**Fig 2:**
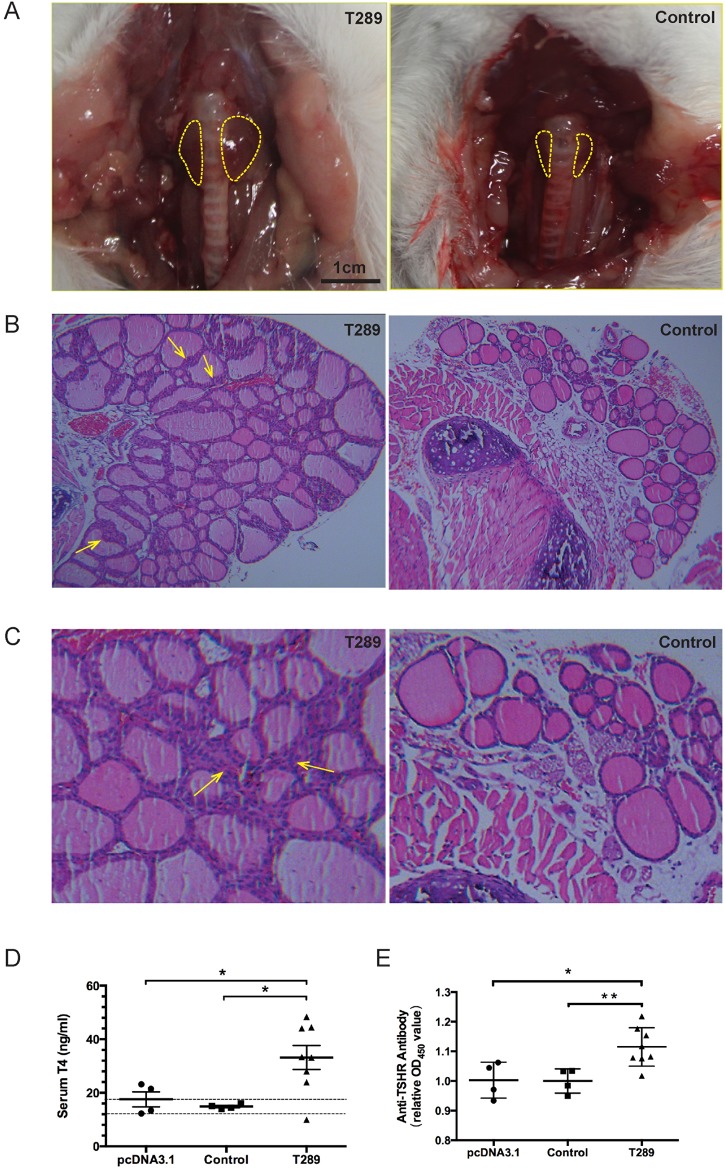
TSHR genetically immunized mice showed typical features of goiter. (A) The immunized mice develop the phenotype of goiter. (B) In H&E staining, the TSHR immunized mice showed typical phenotype of hyperthyroidism (×40). Thyroid epithelial cells are tall and columnar and extend as papillary folds into the follicles (arrowed), indications of hypertrophy and hyperplasia. (C) There is increased number of infiltrated mononuclear cells in the thyroids (arrowed, ×200). (D) The immune mice showed significantly increased level of T4 levels in compared with the age-matched controls. The dotted line indicates mean ±3SD for control mice, *p<0.05. (E) The immune mice showed significantly increased level of TRAb compared with the age-matched controls. The TRAb levels were determined by ELISA and expressed as the fold change of OD450 value compared to control sera, *p<0.05.

In response to the TSHR genetic immunization, it was observed that more than **90%** of the genetically immunized mice **showed obvious** goiter and thyroid hypertrophy (Fig 2A). H&E examination of thyroid glands indicated that there are 80% animals with typical features of hyperthyroid glands (Fig 2B): thyroid epithelial cells are tall and columnar and sometimes extend as papillary folds into the follicles, indications of hypertrophy and hypercellularity [13]. Additionally, we also observed that there was increased inflammatory cell infiltration in the thyroid glands (Fig 2C). Serum T4 levels were consistent with the above results. There are also more than 80% increased T4 levels in the immunized mice in comparison with that of the age-matched control (Fig 2D), and TRAb levels were also significantly increased in the immunized groups (Fig 2E). These above results indicated that we have successfully constructed a Graves’-like hyperthyroidism animal model, which showed some typical pathological features such like elevated T4 levels and significantly increased expression of TRAb.

### TSHR genetically immunized mice concurrently develop the Graves’ ophthalmopathy phenotype

**Fig 3:**
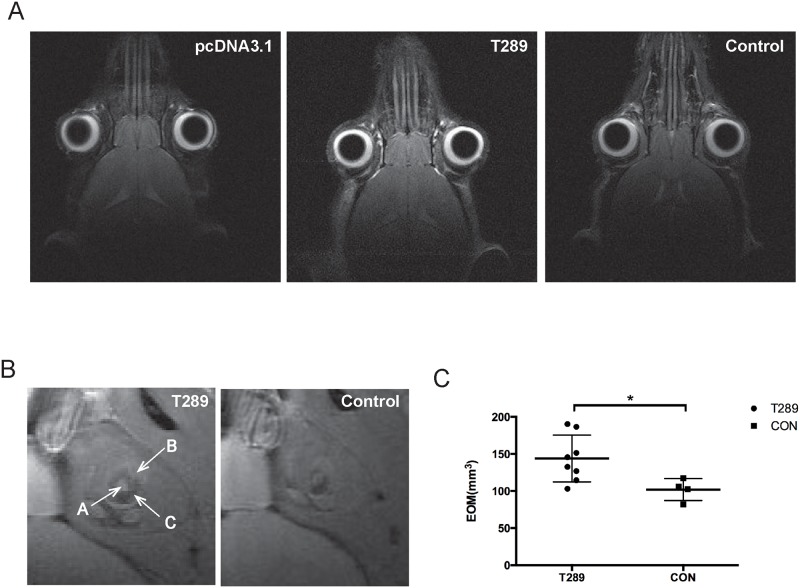
TSHR immunized mice showed enlarged extraocular muscle volume. (A) *In vivo* MRI scaning of the mouse head showed no sign of proptosis, Contiguous T2-weighted MR images of the head at the horizontal level were acquired using a fast-spin-echo (FSE) sequence (0.6mm thick). Judging from the relative position of the eyeball and the outline of the mouse head, no sign of proptosis were observed in the immune mice compared with control. (B) The enlargement of extraocular muscle volume was observed in *in vivo* MRI scaning, Contiguous T2-weighted MR images of extraocular muscles of the right eye were acquired at the sagittal level. The orbital muscles (labeled C) are of lower signal intensity than the adjacent cerebrospinal fluid (in white, labeled A). In turn, the orbital muscles are surrounded by the higher signal intensity of the harderian gland (labeled B). (C) Segmentation analysis by Image J software demonstrated there is a significant increase in the extraocular muscle volume, *p<0.05.

**Fig 4:**
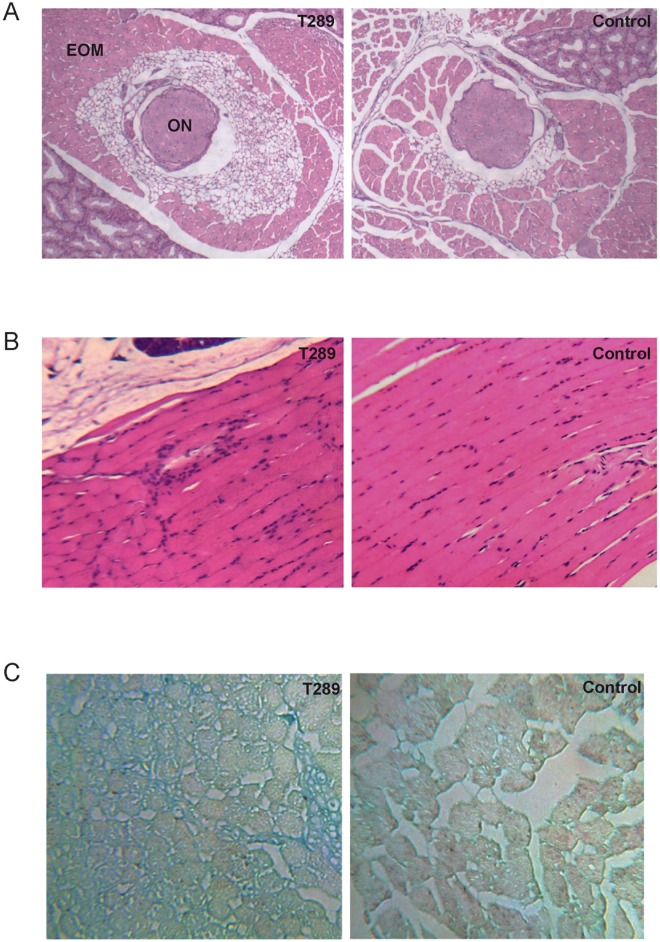
Histological analysis of the orbital tissues from the TSHR immunized mice. (A) H&E staining of the orbital tissue (×40). Mild lymphocytic infiltration was seen between the extraocular muscle around the optic nerve. (B) Adipose tissue around the optic nerve was increased in immunized animals (×40). (C)Alcian blue-stained section of orbital muscle (×400) showed increased production of HA in retrobulbar tissue in immunized mice.

Before the animals were sacrificed, MRI scanning of the orbit has been examined and no signs of proptosis were observed (Fig 3A). However, the orbital muscle volumes of immunized animals calculated by software were significantly increased compared to control mice (Fig 3B and 3C). By histological exanimation, adipose tissue around the optic nerve were observed to increase in 2 orbital tissues (Fig 4A), and we have also observed mild infiltration of immune cells in only one orbital tissue between the extraocular muscles (Fig 4B). Additionally, by using the Alcian blue staining, we also observed that there is increased production of HA in 5 out of 16 orbital tissues between extraocular muscles, indicating that orbital fibroblasts plays a crucial role in the pathogenesis of this diseases by their ability to produce hyaluronic acid (Fig 4C).

## Discussion

*In vivo* murine models of GD provide a valuable resource for the study of pathogenesis and experimental therapies of the diseases. In the past decades, various attempts have been tested to generate the mouse model of GD, however, most of these animal models have certain deficiencies that place limitations on their use [3, 14–16]. In the recent years, it has been recognized that genetic immunization of TSHR seems to be an effective approach to induce the production of TSHR Antibody *in vivo*, which is associated with the development of Graves’ -like hyperthyroidism, either by injection of adenovirus[9, 13] or plasmid[4, 17] vectors.

As one of G-protein-coupled receptors, TSHR has a unique molecular structure, which is comprised of an extracellular A-subunit, a trans-membrane domain and an intracellular B-subunit, held together by a disulphide link and forms a heterodimer [18, 19]. It has been recognized that the soluble A-subunit could be shed from thyroid cell membranes and released into the extracellular space or the bloodstream[20, 21]. After the shedding, the remaining B-subunit on the membrane was unable to induce the TSH signaling pathway [22], therefore, it has been suggested that cleavage of the native TSH receptor and the expression of TSHR-A subunits play more important roles in signal transduction and receptor activation compared with the expression of full length of TSHR. This hypothesis was tested in a previous study that demonstrated *in vivo* over-expression of TSHR A subunit (aa.1-289) could increase the incidence of GD like disease in comparison with a full-length TSHR immunization [9]. In line with this idea, recent studies demonstrated that genetic immunization of TSHR A subunit by using adenovirus vectors could increase the incidence of hyperthyroidism to approximately 65–80%, indicating that over-expression of TSHR A subunit was a very useful and highly reproducible approach to generating the GD like disease phenotype [13].

Initially in many previous studies, the orbital changes were relatively difficult to examine due to shortage of accurate screening technique. In 2013, Johnson KTM et al. [10] have invented a novel technique by removing of the entire mouse orbit and using optic nerve as an anatomical landmark, that increased the sensitivity of retrobulbar detection [10]. Using this technique, Moshkelgosha et al. reported that immunized female BALB/c mice with TSHR A subunit expressing plasmids were able to induce GO like phenotype. This has created a landmark for the generation of a typical murine GO model with the phenotype including the proptosis, orbital inflammation, increased adipose tissue deposition, and the accumulation of fibrosis. But for some unknown reasons, in their study, the thyroid glands mainly manifested typical hypothyroidism with lower T4 value and mainly TSBAb in the sera [8]. Therefore, more efforts are needed to be taken to address the mechanism why this approach produced hypothyroidism rather than more common hyperthyroidism in this process.

As the most important pathogenic antigen, *in vivo* over-expression of TSHR of both human and murine origins has been used to induce GD and GO models in mice, but the success rates varied in different studies[3]. Considering the homology of murine and human TSHR and the higher success rate with human TSHR in previous studies, we chose human TSHR A subunit as our antigen. Our result demonstrated that the genetically immunized mice developed apparent Graves’-like hyperthyroidism phenotype with a relative high prevalence of about 80%. This result indicated that *in vivo* overexpression of human TSHR A subunit gene via genetic immunization is indeed a feasible approach for the induction of murine model of Graves’ disease. By addressing its phenotype, we observed that there are increased serum TSHR antibodies (TRAb) levels in the genetically immunized mice, however, due to technical limitations, we did not perform the functional assay to test the TSH-binding inhibition (TBI) activity or TSAb bioassays, only ELISA was performed to measure total TRAb levels (including non-stimulating TRAb). According to the typical hyperthyroid features of elevated T4 value and pathologically epithelial hypertrophy and hyperplasia, it is reasonable to assume that the result antibodies are predominately TSAbs.

Considering the fact that the *in vivo* gene delivery methodology might influence the efficiency of transfection, in our study, the genetic immunization was optimized via deepening the needle injection to 4-5mm, which ensured the plasmid injection spot were in the midpoint of the line segment between two electrode needles, for the purpose of further increasing the transfection efficiency. This approach was proved to be very successful, since the thyroid morphology and orbital pathology examination all confirmed the successful establishment of the GD animal model.

In addition, with regard to the transfection efficiency, not only the immunization technique matters, the plasmid vector, which carries the antigen, is also very important. In 2007, Kaneda et al. [4]have tried different vectors and found that pBacMam-2 induces higher protein expression. In 2013, Moshkelgosha et al. adopted the vector pTriEx-1.1 neo, to have a successful animal model of GO. However, these vectors are not commonly used and very hard to obtain in some laboratories. Therefore, in the present study, we used the common pcDNA3.1 as a vector, making it much easier to construct the recombinant plasmid and we found that pcDNA3.1 is as good a vector as the other vectors mentioned above.

It has been recognized that an ideal GO mouse model should manifest the following features [23]: 1) Increased serum T4 or decreased serum TSH; 2) The production of TRAb in the serum, preferably TSAb; 3) Alterations of thyroid structure and size; 4) Lymphocytic infiltration in thyroid glands; 5) Clinical manifestations of hyperthyroidism; 6) Gender difference, female animals are more susceptible; 7) Orbital manifestations including extraocular muscles swelling, edema, immune cell infiltration and increased adipose tissue. Here in this study, the TSHR immunized mouse model do exhibit several typical pathological features of GO including extraocular muscle volume expansion based on the MRI scanning result and the mild lymphocytic infiltration in the orbital muscles. The orbital fibroblast (OF) has been considered as the most important pathological cells of GO [2, 24–26], and it has been reported that cultured OF do express functional TSHR and the expression level of TSHR is increased during OF differentiation [27–29]. The OF has been considered as the major cell source for HA synthesis[30], which was activated by cytokines (TGF-β), prostaglandin D2 and growth factors (TSH and IGF-1) [31–33]. Here in our study, by using Alcian blue staining in orbital sections [11, 12], we observed increased release and accumulation of HA (stained positively with Alcian blue) in the extracellular space between muscular fibers, suggesting an occurrence of GO.

Taken together, in this study, we have successfully established a GD murine model with the pathological phenotype of GO simultaneously, indicating that the approach of genetic immunization of TSHR A subunit plasmid is an effective approach for induction of hyperthyroidism and orbitopathy. Hopefully, this approach will provide a very useful tool for further study of immune pathogenesis and novel therapies for GD and GO in the future.

## Supporting information

S1 File Enlargement of extraocular muscle volume. The extraocular muscle volumes are measured by segmentation analysis and the data showed there was a significant increase in the extraocular muscle volume, *p<0.05.
(XLSX)
